# Assessing the Impact of Cool Roofs on Health and Wellbeing: A Scoping Review

**DOI:** 10.1007/s40572-026-00558-2

**Published:** 2026-09-03

**Authors:** Noah Bunkley, Melanie Stowell, Chris Bullen

**Affiliations:** School of Population Health, University of Auckland, Auckland, New Zealand

**Keywords:** Scoping Review, Cool Roofs, Heat, Health, Wellbeing, Housing

## Abstract

**Background:**

Extreme heat poses a threat to the health and wellbeing of millions of people worldwide living in climate-vulnerable housing. Cool roofs are a potential passive-cooling housing retrofit solution that is affordable and scalable to reduce heat exposure and improve community health and wellbeing.

**Objective:**

To conduct a scoping review to systematically identify and map the research addressing the effect of residential cool roofs on health and wellbeing, identify knowledge gaps, and inform the design of future empirical studies.

**Design:**

Six online databases were searched from 1980 to July 2025 to identify relevant papers. We selected 14 publications that compared cool roofs with non-cool roofs, measured their effects on health and wellbeing, and summarised the data in a narrative synthesis.

**Results:**

All publications were modelling studies, with the majority (71%) examining the effects of cool roofs on urban heat island mitigation and their impact on health. Most (93%) of the assessed populations were in high-income countries. Estimates of the projected health benefits of cool roofs ranged widely across studies, depending on the study context, methodology, and outcomes.

**Conclusions:**

While our scoping review identified modelling studies highlighting the potential benefits of cool roofs, the dearth of publications assessing their use in low-resource settings and the lack of empirical evidence underscore the need for further research to determine their effectiveness in structurally vulnerable communities.

**Supplementary Information:**

The online version contains supplementary material available at https://doi.org/10.1007/s40572-026-00558-2.

## Introduction

Climate change-induced heat threatens the health and wellbeing of structurally vulnerable communities worldwide, and effective, reliable, affordable, and scalable adaptation interventions are urgently needed. Several studies have estimated the projected increase in heat exposure due to climate change across many regions worldwide under varying carbon-emission and economic-development scenarios [1–4]. Even with global warming kept within 2° C, by 2050, exposure to dangerous levels of heat and humidity is projected to occur for one-quarter to one-half of all the days in each year in the tropics, threatening to place millions of people at risk from extreme heat events (EHE) and extreme heat exposure [5]. Exposure to extreme heat increases morbidity and mortality, with short-term exposure resulting in heat stress and heat stroke, and long-term exposure increasing the risk of a range of health conditions, including increased cardiovascular disease, kidney failure, respiratory disease and mental health symptoms [6–11]. Currently, 0.9% of all deaths globally are attributable to heat [12], with an increase of 63.2% for heat-related mortality since the 1990s, translating to an average of 546,000 deaths per year between 2012 and 2021 [13]. The impacts of heat are unevenly distributed globally and depend on geography and individual and community-level adaptive capacity. Adaptive capacity reflects an individual’s access to the social determinants of health, such as adequate income, housing and education, meaning heat will have a greater impact on those living in low-resource communities, worsening global health inequities [14].

Cool roofs are one proposed solution to equitably reduce indoor air temperatures and the Urban Heat Island (UHI) effect in communities most impacted by extreme heat through increasing building-level and urban-level roof solar reflectance and thermal emittance [15]. Typically, roofs are dark and absorb over 80% of sunlight, conducting heat into the building envelope and trapping it in the local environment. The absorbed solar energy through urban roofs contributes to the UHI effect, which results from the increased heat generation from vehicles and buildings and heat absorption from dark, paved surfaces that typically cover urban areas [16, 17]. Cool roof coatings are low-cost, passive building modifications that can reduce peak indoor air temperatures and reduce UHIs [18, 19]. Unlike air conditioning, which can increase urban heat load, cool roofs can also reduce building energy consumption and associated air pollutant emissions [20, 21]. The effectiveness of cool roofs in reflecting light is often measured by their albedo, the fraction of light they reflect, ranging from 0 (completely black, absorbing all light) to 1 (completely white, reflecting all light).

Several reviews have examined the evidence of the effectiveness of cool roof technology in reducing indoor air temperatures and improving energy use, noting the benefits at the building and city scale regarding improved thermal comfort, increased energy efficiency, attenuated UHIs, and reduced air pollution [22–25]. However, the quantity and quality of information in the literature linking cool roof use on residential homes to improved health and wellbeing remain unclear. Studies that assess the health impacts of cool roofs are an essential data resource to test assumptions about the potential for health improvements in real-world settings, particularly to inform climate change adaptation strategies in low-resource communities. For these reasons, we have conducted a scoping review to systematically identify and map the research addressing the effect of residential cool roofs on health and wellbeing, to identify knowledge gaps, and to inform the design of future empirical studies.

## Methods

Scoping reviews are a form of systematic evidence synthesis used to map key concepts within a research field, explore the breadth or extent of the literature and define the conceptual boundaries of a topic primarily to inform future research [26, 27]. Unlike systematic reviews, scoping reviews do not report synthesised results from multiple evidence sources but instead aim to provide an overview of the evidence. Our scoping review was guided by the Preferred Reporting Items for Systematic Reviews and Meta-Analyses extension for Scoping Reviews (PRISMA-ScR) Checklist [28].

### Eligibility Criteria

#### Types of Studies

We included before-and-after, prospective and retrospective cohort, case-control, analytical cross-sectional, controlled, uncontrolled, randomised (including cluster-randomised), and non-randomised studies of the health and social effects of cool roofs in the review. Intervention studies reporting quantitative or qualitative data, or both, were also included. To determine the extent of the literature assessing cool roofs in practice, experimental, quasi-experimental, observational and modelling studies were included. Qualitative studies, reviews and conference abstracts were excluded.

#### Types of Participants

Our review did not exclude any participants based on family type, socio-economic status, or other equity indicators such as race or ethnicity, occupation, education, or religion. Studies from any region, climate type, and country income categorisation were eligible for inclusion. Outcomes for both adults and children were eligible for inclusion in the review.

#### Types of Housing

All physical house types that are static (that is, not caravans or houseboats) were eligible for inclusion. Mobile homes and houseboats were not included because these types of housing may be exposed to a range of different conditions and are more likely to serve different purposes, for example, recreational purposes. Static permanent housing included residential establishments providing permanent accommodation and sheltered housing.

#### Types of Intervention

Cool roof intervention was defined as the installation or application of any cool roof materials that increased the albedo of resident roofs, for example, liquid-applied membranes (LAM), shingles, coatings, stone/rock, metal, or tile.

#### Types of Outcomes

Studies were included if they investigated changes in health, illness, or wellbeing-related outcomes among residents either before and after, or with and without cool roofs. Outcome measures included any measure that could be interpreted as a direct measure of health or mental and physical illness, general measures of self-reported wellbeing, and quality-of-life measures such as comfort.

### Information Sources

To identify potentially relevant documents, the following bibliographic databases were searched from 1980 to July 2025:

- MEDLINE (MEDical Literature Analysis and Retrieval System OnLINE) (Ovid) (1947-present).
- EMBASE (Excerpta Medica dataBASE) (Ovid).
- Scopus.
- CINAHL (Cumulative Index to Nursing and Allied Health Literature) Complete (Ovid).
- Web of Science.
- Google Scholar.

We searched these databases because they contain literature relevant to health and wellbeing.

### Search

The original search question was divided into three concepts for the purposes of searching, including cool roof, heat and housing. The number of concepts searched depended on the type of database. We defined a basic set of search terms covering each concept. See the appendix for example search phrases. 

### Selection of sources of evidence

The results were screened by the lead author (NB), using rayyan.ai [29]. The co-author (MS) screened 10% of the results to assess congruity [30]. We selected articles with titles and abstracts deemed relevant for full-text review. Full-text articles that were relevant were included for analysis. Uncertainties in study selection and data extraction were resolved by consensus and discussion within the research team. We searched the reference list of identified reports and articles selected for full-text review for additional sources.

### Data Charting Process

We developed a data-charting form to determine which variables to extract. Data from the included articles was then charted, and the data-charting form was continuously updated through an iterative process.

### Data Items

We extracted data on article characteristics (e.g. authors, year of publication, funder), study characteristics (e.g. location, study design, timeframe), participants (e.g. demographics, housing typology), cool roof intervention, and outcomes (e.g. mortality, morbidity, quality of life, sleep, physical activity, productivity).

### Quality

Methodological quality and model credibility were appraised using an adapted framework based on the International Society for Pharmacoeconomics and Outcomes Research–Society for Medical Decision Making (ISPOR-SMDM) Modeling Good Research Practices guidance [31]. Studies were evaluated across six domains for their ability to address the research question: model structure, assumptions, data inputs, validation, uncertainty analysis, and transparency. Each domain was qualitatively rated as high, moderate, or low based on predefined criteria developed a priori. See the appendix for the domain criteria and individual study quality appraisal.

### Synthesis of Results

We grouped the studies by setting and study design and summarised the outcomes for each group, along with the measures used and broad findings. Where we identified another review article, we searched its bibliography for articles that potentially met our inclusion criteria and included them in our synthesis.

### Use of Artificial Intelligence Tools

No generative artificial intelligence or large language model tools were used in the conception, design, conduct, data extraction, analysis, or writing of this review.

## Results

### Selection of Sources of Evidence

**Fig. 1:**
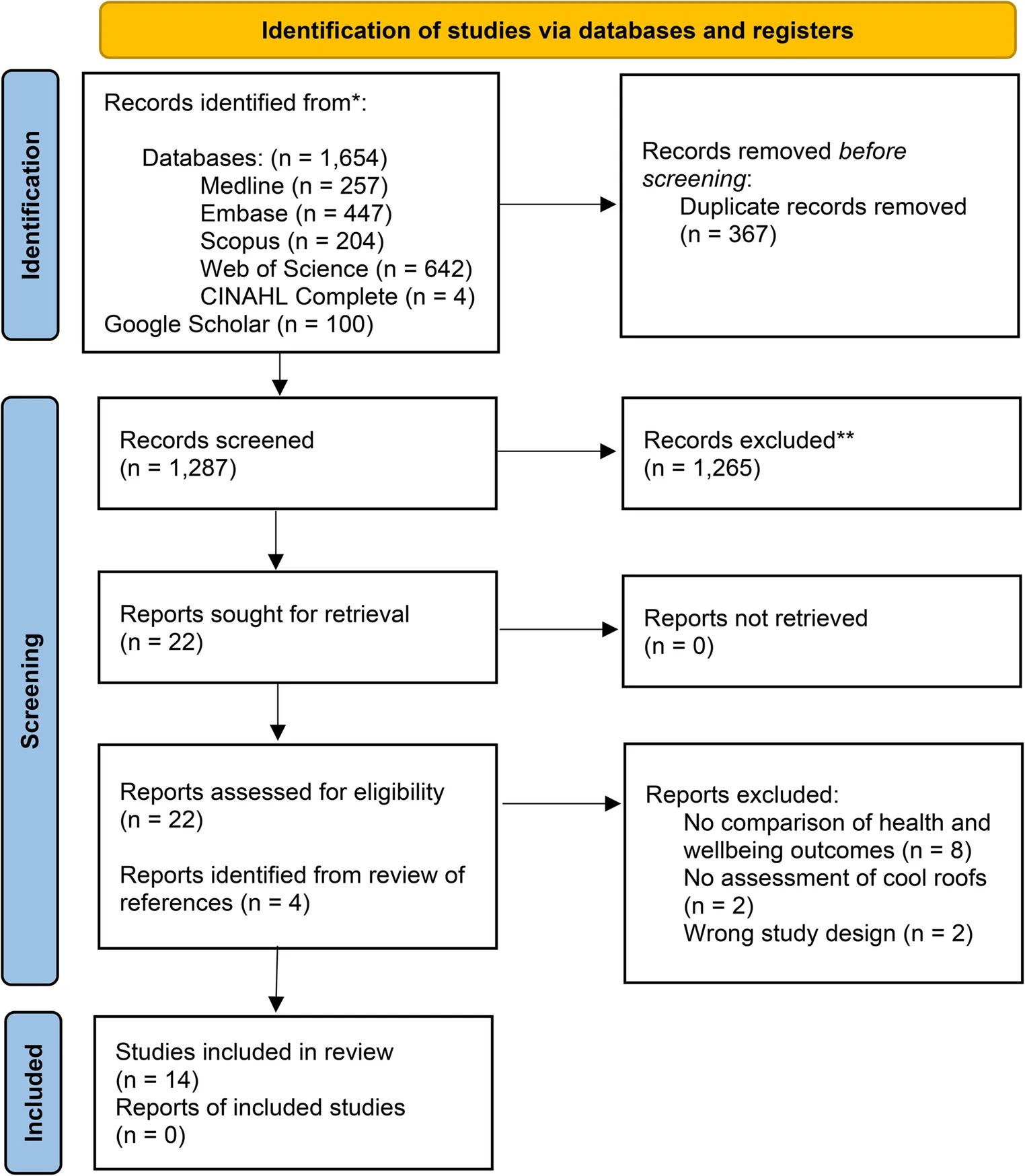
PRISMA diagram showing records identified, screened and included in the review

After screening 1,287 titles and abstracts and 26 full-text documents, 14 papers fulfilled our eligibility criteria (Fig. 1). Initial interrater agreement for the 10% subset was 97.7% prior to discussion and consensus within the research team. Eight documents were excluded because they measured the wrong outcome and did not contain an assessment of health and wellbeing, two were excluded because they measured the wrong comparator and did not contain an evaluation of cool roofs, and two were excluded because they were the wrong study design, with one being a protocol publication and the other a review article. Four reports were identified through scanning reference lists.

### Characteristics of Sources of Evidence

All documents were published since 2012 (Table 1) and predominantly contained analyses of populations based in just three countries: the USA (43%), the UK (14%) and Germany (14%). Only one paper assessed informal settlements in LMICs (low- and middle-income countries) (7%). All papers were classified as modelling studies, that is, research that uses models to represent and analyse real-world systems [32]. Ten studies (71%) modelled the impact of cool roofs on UHIs, two (14%) modelled the effects on indoor conditions, and two (14%) modelled both indoor and outdoor conditions. Where studies addressed UHIs, most measured the effects across the entire population within their study settings. For the two studies that addressed indoor conditions, each identified the specific housing typology used in their research. Equity and vulnerability were incorporated inconsistently, with one study (7%) estimating avoided mortality by age, income, and race across census tracts. Other studies considered vulnerability through age-stratified mortality estimates [33, 34], subgroup exposure profiles [35], or focused on high-risk settings such as informal settlements and low-income households [36]. Funding declarations varied across studies, with most reporting public research, government, or institutional support; two studies reported municipal or industry-linked support; two stated that no specific grant funding was received; and one did not report a funding source.

### Results of Sources of Evidence

Twelve studies (86%) published the albedo measure used in their models for their cool roof or increased albedo scenarios (Table 2). Four studies (29%) included increased footpath and/or road albedo alongside increased roof albedo in their models. Ten studies (71%) reported on the impact of cool roofs on mortality, three (21%) on heat-stress hours (defined as time spent above a temperature threshold), and two (14%) on thermal comfort. Reporting of cool roof relevant results was inconsistent across the studies, with two studies (14%) reporting on the impact of cool roofs in combination with other adaptation measures and two studies (14%) failing to report on the specific magnitude of effect in the body of the text, but contained graphs that could be used to estimate a magnitude of effect.

Estimates of the effect of cool roofs on health and wellbeing ranged widely. Studies that reported relative risk reductions in mortality estimated the effect to be between 0.21% (based on summer-avoided heat-related deaths for an urban roof albedo of 0.88) and 58% (based on an effective irradiation reduction of 20%). Two studies reported increases in cold-related mortality of 0.096% and 0.23% in winter. For those that reported on heat stress hours, the magnitude of effect ranged from a daily reduction in strong heat stress of two hours, with no change in total duration of heat stress based on modelled impacts on UHI in Berlin, to a decrease of heat stress incidents of 91% based on indoor temperatures in Jakarta. For those that reported on thermal comfort, the magnitude of effect estimates ranged from no effect on outdoor thermal comfort (based on a UHI assessment of a university campus in Rome) to increases in non-airconditioned indoor thermal comfort time of 9.3% (based on a wooden, urban house model in Japan).

### Quality of Sources of Evidence

Across the included studies, methodological credibility was variable. Studies were strongest when they specified a clear causal pathway from roof albedo modification to modelled ambient or indoor exposure, and then to health-relevant outcomes, and included validated, region-specific exposure-response functions [33, 34]. Several studies had strong models but provided only indirect evidence for cool roofs specifically, because roof albedo changes were combined with pavement albedo, vegetation, irrigation, or broader land-cover scenarios, or they did not include direct assessments as to how cool roof changes led to their measured outcomes, limiting attribution of health benefits to cool roofs alone [35, 37–41]. Evidence was also weaker for outcomes such as thermal comfort or heat-stress proxies, such as Wet Bulb Globe Temperature, Universal Thermal Climate Index (UTCI), Mediterranean Outdoor Comfort Index (MOCI), or indoor overheating hours, rather than mortality, morbidity, symptoms, or self-reported wellbeing [36, 39, 42–44]. Common limitations across the evidence base included limited validation against observed cool-roof implementation data, incomplete propagation of uncertainty from physical climate or building models through to health estimates, and limited modelling of indoor exposure.

Several assumptions in the model pathways limit the credibility of the projected health estimates. The translation from roof surface temperature reduction to ground-level ambient temperature reduction involves assumptions about atmospheric mixing, building geometry and convective heat transfer that introduce substantial uncertainty. No studies validated their ground-level temperature reductions against observed temperature data following actual cool roof installation. One study directly addressed the impact of cool roofs at ground level and found that cool roofs had little effect on pedestrian-level MOCI because roofs were too distant from the human exposure height [42]. Several studies acknowledged that health-relevant heat exposure may be more closely related to indoor or personal exposure than to outdoor ambient temperature, yet applied exposure–response functions derived from outdoor temperature data because population-scale indoor exposure data were unavailable or beyond the scope of regional weather models [33, 34, 45]. Some studies modelled indoor conditions or personal exposure pathways, but either retained outdoor-temperature-based mortality models or reported indoor heat-stress/comfort proxies rather than applying indoor-derived health exposure–response functions [35, 36, 39, 44].

**Table 1:** Characteristics of sources of evidence

Reference	Aim	Study design	Setting	Population	Health outcome
Buchin et al. [37] 2016	To quantitatively assess how different UHI mitigation strategies—including cool roofs, cool pavements, vegetation, and façade or roof greening—can reduce heat-related health risks in urban areas.	Modelled impact on indoor heat exposure and UHI	Berlin, Germany	Over 65-year-olds	Mortality
He et al. [45] 2020	To quantify the impact of green and cool roofs on temperatures in both summer and winter, and to assess the potential health implications of those temperatures.	Modelled impact on UHI	Greater Boston area and New England area, USA	Total population	Mortality
Kalkstein et al. [38] 2020	To quantify how increasing urban reflectance using reflective roofing products would lessen the intensity of EHEs and save lives in Boston and Chicago.	Modelled impact on UHI	EHEs in Boston (1994, 1999, 2002 and 2007) and Chicago (1988 and 2012), USA	Total population	Mortality
Liu et al. [44] 2023	To investigate the effects of density and UHI countermeasures on energy consumption and indoor thermal comfort in densely built wooden house areas.	Modelled impact on indoor thermal comfort	Yokohama City, Japan	A single detached household in a densely built wooden house area	Thermal comfort
Macintyre and Heaviside [33] 2019	To assess the potential effect of cool roofs on local ambient temperatures and the subsequent impact on heat-related mortality.	Modelled impact on UHI	West Midlands (summer of 2006 and EHEs in 2006 and 2003), UK	Total population	Mortality
Macintyre et al. [34] 2021	To estimate the impact of cool roofs on cold-related mortality in winter.	Modelled impact on UHI	Birmingham and West Midlands (winter 2009/10 and 2080 under high emissions scenario), UK	Total population	Mortality
Nutkiewicz et al. [36] 2022	To evaluate the exposure to heat stress in informal settlements and the effect of passive cooling solutions to achieve adequate thermal comfort.	Modelled impact on indoor heat stress	Bangalore, Jakarta, Mombasa and Mumbai	Houses in informal settlements	Heat stress
Sailor et al. [35] 2021	To explore the indoor and outdoor exposure conditions for urban residents and the implications for adverse health outcomes.	Modelled impact on combined indoor and outdoor environments	Los Angeles (EHEs in 2006 and 2009), USA	Total population	Mortality
Salata et al. [42] 2017	To examine the influence of the mitigation strategies on outdoor comfort.	Modelled impact on UHI	Campus of the Sapienza University of Rome, Italy	Total population	Comfort
Santamouris et al. [39] 2020	To calculate the impact of local overheating on the energy demand, indoor environmental quality, vulnerability and survivability and heat-related mortality and morbidity using multiyear energy, environmental and health data in eight mitigation scenarios	Modelled impact on UHI	Greater Sydney, Australia	Total population	Heat stress and mortality
Stone et al. [40] 2014	To estimate changes in heat-related mortality resulting from future year climatic conditions and the influence of alternative heat management strategies on health outcomes.	Modelled impact on UHI	Phoenix, Atlanta and Philadelphia (2050 warm season), USA	Total population	Mortality
Susca [46] 2012	To determine the effect of urban rooftop albedo on human health.	Modelled impact on UHI	Columbia University and Con Edison areas, New York City, USA	Total population	Mortality and DALY reduction
Vargo et al. [41] 2016	To evaluate the effectiveness of planning strategies to lower summer ambient temperatures and thereby reduce mortality for different races, ages, and income levels in the three metropolitan areas.	Modelled impact on UHI	Phoenix, Atlanta and Philadelphia (2050 warm season), USA	Total population. Subgroup analysis by age, income and race.	Mortality
Wang et al. [43] 2022	To evaluate the effectiveness of cool and green roofs in improving human thermal comfort.	Modelled impact on UHI	Berlin (EHE 2014), Germany	Total population	Heat stress

*Abbreviations*: *EHE *Extreme heat event, *UHI *Urban heat island, *DALY *Disability-adjusted life year

**Table 2:** Results of sources of evidence

Reference	Comparison	Control	Exposure-response function source	Exposure pathway	Main Health-Related Cool Roof Findings
Buchin et al. [37] 2016	Not reported	Not reported	Self-derived; regression of all-cause mortality on modelled indoor air temperature (threshold 29 °C).	Combined	A 20% reduction in effective irradiation would reduce the number of excess deaths by 58%. No direct comparison of cool roofs versus the control was reported, and no description of how cool roofs specifically contribute to a reduction in effective irradiation was provided.
He et al. [45] 2020	Roof albedo of 0.88 on all roofs in urban areas	Roof albedo of 0.13 on all roofs in urban areas	External [47]; +1% mortality per °C above 16.39 °C (summer), + 0.6% per °C below 2.35 °C (winter); derived from New England, USA.	Outdoor	In summer, cool roofs would reduce heat-related deaths by 0.210% (95% CI = 0.126%–0.315%). In winter, cool roofs would increase the cold-related deaths by 0.096% (95% CI = 0.048%–0.144%).
Kalkstein et al. [38] 2020	REFL1 = Roof albedo and road albedo of 0.3 REFL2 = Roof and road albedo of 0.4	Roof and road albedo 0.15	Self-derived; synoptic regression of daily excess mortality on oppressive-air-mass days	Outdoor	For Boston, the total baseline mortality for the evaluated events was about 122 deaths. This would have been reduced to 112 (8.1% reduction) for the REFL1 scenario and 109 (9.2% reduction) for the REFL2 scenario. For Chicago, the total baseline mortality for the evaluated events was about 305 deaths. This would have been reduced to 301 (2% reduction) in the REFL1 scenario and to 265 (13% reduction) in the REFL2 scenario.
Liu et al. [44] 2023	Roof albedo of 0.6	Roof albedo of 0.2	None; indoor thermal-comfort hours (SET* 22.2–25.6 °C comfort band [48]).	Indoor	Cool roofs would increase non-air-conditioned indoor thermal comfort time by an average of 9.3%. There would be no effect on indoor thermal comfort during hours of air-conditioning use; however, cool roofs would reduce total air-conditioned hours.
Macintyre and Heaviside [33] 2019	Roof albedo of 0.7	Roof albedo of 0.2	External [49]; 2.5% mortality per °C above 17.7 °C daily mean; derived from UK.	Outdoor	Cool roofs implemented across the whole city would offset 18% of seasonal heat-related mortality associated with the UHI (corresponding to 7% of total heat-related mortality). During heatwave periods, covering all buildings with cool roofs would reduce overall heat-related mortality by approximately 8% and offset 25% of that attributable to the UHI.
Macintyre et al. [34] 2021	Roof albedo of 0.7	Roof albedo of 0.2	External [49]; +1.8% mortality per °C below 11.7 °C daily mean (age-stratified); derived from UK.	Outdoor	Cool roofs in summer could offset 34 deaths in the 2080s. Cool roofs would have a negligible effect on cold-related mortality in winter (4 additional cold-related deaths; 0.23% of overall cold-related deaths).
Nutkiewicz et al. [36] 2022	Not reported	Not reported	None; indoor heat-stress threshold (WBGT > 35 °C for > 4 h [50]).	Indoor	Cool roofs would mitigate between 44% and 91% of heat stress incidents and result in an absolute reduction of heat stress hours per year of 1,678 in Bangalore, 3,470 in Jakarta, 2,444 in Mombasa, and 2,861 in Mumbai.
Sailor et al. [35] 2021	Roof albedo of 0.45 and pavement albedo of 0.35	Roof albedo of 0.17 and pavement albedo of 0.1	Self-derived; synoptic regression of daily excess mortality on oppressive-air-mass days.	Combined	Cool roofs would have led to a 13% decrease (8 lives) in heat-induced mortality during the 2006 EHE and a 50% decrease (10 lives) in heat-related mortality during the 2009 EHE.
Salata et al. [42] 2017	Roof albedo of 0.66	Roof albedo of 0.35	External [51]; MOCI mapped to a borrowed mortality threshold (+ 3.12% per °C above apparent temperature 29.4 °C); derived from 15 European cities, > 75 year.	Outdoor	No change in outdoor comfort associated with cool roof use.
Santamouris et al. [39] 2020	Roof albedo of 0.66 and pavement albedo of 0.4	Roof albedo of 0.15 and pavement albedo of 0.08	Self-derived; regression of daily health anomalies on daily maximum temperature (morbidity + 1.1% per °C; mortality + 5.3% per 2 °C).	Outdoor	No reporting on the impact of cool roofs alone. Mitigation technologies would reduce overheating hours above 30 °C by between 5% and 21% and above 32 °C by 5% and 35%, depending on the mitigation technology chosen.
Stone et al. [40] 2014	Roof albedo of 0.9 and pavement albedo of 0.45	-	External; three US epidemiological coefficients combined in BenMAP: minimum temperature [52], apparent temperature [53], and an additive per heat-wave-day term [54]; derived from multi-city US samples.	Outdoor	No reporting on the impact of cool roofs alone in the body of the text. However, a graph indicates cool roofs and pavement albedo enhancement would result in 20–70 fewer deaths per year in each city.
Susca [46] 2012	Roof albedo of 0.9	Roof albedo of 0.32	External [55]; New York City heat-index/natural-cause mortality regression, converted to DALYs; derived from New York City.	Outdoor	Cool roofs in the Con Edison building area would result in a decrease in mortality relative risk corresponding to about 45 avoided deaths each year, most of which would be people between 45 and 84 years of age. The decrease in annual mortality from natural causes would correspond to about 751 DALYs avoided annually. Cool roofs showed no effect on mortality reduction in the Columbia University Area.
Vargo et al. [41] 2016	Roof albedo of 0.9 and pavement albedo of 0.45	Roof and pavement albedo of 0.15	External; two US epidemiological coefficients combined in BenMAP: minimum temperature [52] and an additive per heat-wave-day term [54]; derived from multi-city US samples.	Outdoor	Cool roofs would yield greater benefits for older people and low-income communities (Atlanta only). There were variations in impact by race, with greater benefits for non-white individuals in Atlanta and for white individuals in Phoenix. No reporting of the mortality rate reduction due to cool roofs was presented. However, a graph indicated that increasing albedo provides an average avoided mortality rate of approximately 0.2–1.2/100,000.
Wang et al. [43] 2022	CR0.5 = roof albedo of 0.5 CR0.85 = roof albedo of 0.85	Roof albedo of 0.163	None; outdoor UTCI thermal-comfort index and strong-heat-stress-hour reduction.	Outdoor	Cool roofs (CR0.85) would decrease the daily duration of strong heat stress (32 °C < UTCI ≤ 38 °C) from 7 h to 5 h during EHE, but the total duration of heat stress (moderate (26 °C < UTCI ≤ 32 °C) and strong) and the duration of no thermal stress remain the same.

*Abbreviations*: *BenMAP *Environmental Benefits Mapping and Analysis Program, *CI *Confidence interval, *CR0.5*/*CR0.85 *Cool roof scenarios with roof albedo of 0.5 and 0.85, respectively, *DALY *Disability-adjusted life year, *MOCI *Mediterranean Outdoor Comfort Index, *REFL1*/*REFL2 *Reflective roof and road albedo scenarios, *SET* Standard New Effective Temperature, *UTCI *Universal Thermal Climate Index, *WBGT *Wet-bulb globe temperature

## Discussion

### Summary of Evidence and Interpretation of the Findings

We identified 14 studies addressing the impact of cool roofs on health and wellbeing across various time and place settings published between 2012 and 2023. Overall, the identified studies supported the use of cool roofs to reduce heat-related mortality, reduce heat stress, and increase indoor thermal comfort, but were of mixed methodological quality and therefore their effect estimates should be interpreted cautiously.

All 14 studies were modelling studies that used three broad analytic approaches: seven applied previously established exposure-response functions from the heat-mortality epidemiological literature to modelled temperature or thermal-comfort changes from varying albedo scenarios; four derived their own exposure-response or synoptic mortality functions from local data; and three applied no health exposure-response function, instead reporting thermal-comfort or heat-stress indices as health-relevant proxies. These analytic approaches provide policy-relevant estimates of the potential health benefits of cool roofs under specified modelling assumptions and are useful for comparing plausible intervention scenarios and estimating the magnitude of avoided heat-related mortality or morbidity [56]. However, their conclusions depend on the validity of the assumed pathway from roof albedo change to health-relevant exposure and a range of underlying assumptions that may not hold true in reality. They do not provide empirical evidence of effectiveness and should be interpreted solely as modelled plausibility and potential impact rather than as direct causal evidence.

Cool roofs can influence health through two distinct causal chains, which the literature rarely separates explicitly and which carry different evidence bases and uncertainties. In the outdoor pathway, increased roof albedo may reduce sensible heat flux and the UHI effect, thereby reducing heat exposure at the city level. However, this heat difference must propagate downward through the urban canopy layer to reach pedestrian height, where people are exposed, and can attenuate to near zero in high-rise settings, as Salata et al. found at street level on a built-up campus [42]. In the indoor pathway, increased roof albedo acts more directly by reducing heat conducted through the building envelope and individual heat exposure at the building level. No study in our review produced a cool-roof-attributable avoided mortality or morbidity estimate through the indoor pathway. The apparent exception is Buchin et al. [37], which derived its own exposure-response function by regressing all-cause mortality on modelled indoor air temperature; however, the study reported no direct comparison of cool roofs against the control scenario and gave no description of how cool roofs specifically contributed to the modelled reduction in effective irradiation, so its estimate is not attributable to cool roofs specifically. Every study that produced a cool-roof-attributable estimate of avoided mortality did so through the outdoor pathway, using exposure-response functions derived from outdoor temperature. The indoor pathway was represented only by thermal-comfort or heat-stress proxies, or, in studies that modelled indoor conditions alongside outdoor ones, by indoor exposure metrics that were never linked to a health exposure-response function.

The sparse incorporation of indoor heat exposure into most cool roof and health models is likely a symptom of the literature’s general inattention to low-resource communities. Houses in these communities are often constructed with poorer-quality materials and have limited access to energy, preventing active cooling such as air conditioning, leading to indoor temperatures that often exceed ambient temperatures [57]. Roof albedo changes in low-resource settings may produce a comparatively larger direct indoor temperature response compared to well-insulated buildings in higher-resource settings, where the outdoor pathway may dominate. Exposure misclassification from using outdoor temperatures rather than personal heat exposure estimates may yield inaccurate epidemiological findings, given differences between indoor and outdoor environments [58]. As identified in this scoping review, the indoor causal chain most relevant to the structurally vulnerable communities that stand to benefit most from cool roofs is precisely the one for which current evidence is weakest; therefore, for models to credibly estimate the benefits of cool roofs in low-resource populations, they need to incorporate more robust indoor heat exposure-response pathways.

There was a notable lack of studies addressing communities in LMICs and low-resource populations, despite these structurally vulnerable populations showing the greatest need for passive housing heat adaptations and being likely to benefit the most from cool roofs. Only one study, Nutkiewicz et al., provided a modelled impact assessment of cool roofs in LMICs and showed a sizable reduction in indoor heat-stress incidents [36]. Similarly, only one study, Vargo et al., incorporated a subgroup analysis by age, income and race, finding that older populations benefited consistently, but that the distribution of benefit by income and race depended on urban form [41]. Future research should prioritise populations disproportionately exposed to indoor heat due to housing quality and income constraints, and assess the distributional equity effects of cool roof interventions within each study setting [59].

Our scoping review highlights the variation in roof albedo levels used in modelling studies. There appeared to be no consensus on the appropriate albedo level for control and cool roof houses, with values ranging from 0.13 to 0.35 and 0.3 to 0.9, respectively. There is likely significant variation in albedo in both control and roof houses, depending on the setting [60], or the assumed age of the cool roofs as the reflective material fades with time or is covered with debris such as dust or moss [61]. Clear guidelines for modelling input variables, such as albedo levels, could help reduce heterogeneity and improve the comparability of results across studies.

Many of the models explored multiple cooling interventions within the same model and sometimes included increasing footpath albedo alongside roof albedo in their increased albedo scenarios, yet reported only the combined intervention effects. Clarity in the individual reporting of each model output, with explicit magnitude of effects and uncertainty parameters included in the body of the text, would assist readers in identifying the specific results of each modelled scenario [32]. While graphs are useful for interpretation, they are difficult to locate accurate measurements from and therefore draw conclusions on the magnitude of the effect [32]. Future modelling studies should consider individual and combined reporting of each scenario assessed, with the magnitude of effects and confidence intervals reported in the manuscript to assist in interpreting results for readers interested in only a specific intervention.

### Comparison with the Literature

Several recent reviews have examined adjacent aspects of the urban heat and cooling literature, including the health impacts and mitigation of urban heat islands, community-based heat adaptation interventions, and the cooling performance of roof-based mitigation strategies [22–25, 62–65]. However, these reviews do not specifically evaluate cool roofs through a health and wellbeing lens. Broader UHI-health reviews synthesise evidence on heat exposure, morbidity, mortality, equity, and urban mitigation generally, but cool roofs are considered only as one of several physical mitigation strategies, and they do not consider the impacts of cool roofs on indoor environments [65]. Reviews of community-based heat adaptation interventions focus largely on behavioural, educational, and community preparedness strategies rather than built-environment interventions [64]. Conversely, reviews of cool roofs and roof mitigation strategies have primarily assessed thermal performance, urban temperature reduction, building energy demand, or material properties, with limited assessment of their effects on health outcomes [22–25, 62, 63]. The unique contribution of our review is its health-specific synthesis of cool roof interventions, showing that while cool roofs are supported by a substantial temperature and energy literature, the evidence that they improve health and wellbeing remains largely modelled, indirect, and unevenly distributed across settings, with a particular lack of empirical studies in low-resource and heat-vulnerable communities.

### Recommendations for Future Research

Our findings indicate a scarcity of empirical research on the real-world implications of cool roof application. All identified studies were modelling studies that, while helpful for hypothesis generation, rely on assumptions and simplifications that may not reflect the complexities of real-world settings and involve uncertainties in parameter and model structure selection, resulting in findings that may not reflect reality [66]. Although suggestive of potential health and wellbeing benefits, the included modelling studies in this review highlight the need for high-quality confirmatory experimental studies in real-world settings to generate empirical data to validate the models’ findings.

The included studies reflect the tradition in heat–health research to prioritise mortality as the primary endpoint [7, 67–69]. While these outcomes are policy-relevant and comparable across settings, they capture only the most severe manifestations of heat exposure. A substantial proportion of the health burden associated with heat is subclinical, cumulative, and expressed through changes in physiological regulation, daily functioning, and wellbeing [70]. Future research must therefore adopt a broader, multi-domain approach to outcome selection that reflects the full spectrum of heat-related health impacts to detect meaningful differences in heat interventions. Given the wide range of consequences of heat exposure on health and wellbeing [6–11], empirical research should assess a range of outcomes depending on the study context and duration of follow-up, including clinical endpoints such as all-cause mortality, hospital admissions, and disease incidence, physiological intermediates, such as cardiovascular and heat stress indicators and patient-reported outcomes such as sleep quality, thermal comfort, and heat-related symptoms.

The methodology of future empirical studies should focus on explanatory designs, such as cluster-randomised controlled trials (cRCTs), to assess the effectiveness of cool roofs in altering resident health and wellbeing and to expand on the potential identified in the modelling studies. cRCTs are recommended as clustering is necessary because the intervention must act at the building (cluster) level, and randomised trials are the gold standard for determining causation [71]. Ongoing studies, such as a cool roof implementation trial in rural Africa [72], the REFLECT study in Mexico, Burkina Faso, India, Fiji and Niue [73], and other heat adaptation trials as part of the HeatNexus cohort [74], are currently in the process of developing empirical evidence but more research is needed to explore the effectiveness of cool roofs in a variety of settings. There are challenges in conducting empirical studies of community-based cool roof interventions, including outcome selection and measurement methods, blinding difficulties with visible roof modifications, and contamination in cluster designs, but evidence is needed nonetheless. Understanding the causal effects of cool roofs on health and wellbeing is essential for policymakers and funders to support effective climate change and heat adaptations, particularly in structurally vulnerable communities, and to advance global environmental justice.

### Limitations

Our scoping review had several limitations. First, the review lacked a protocol registered prior to its undertaking; therefore, despite our best efforts to limit post-hoc decision-making, there is a risk of bias and reduced methodological transparency.

Second, to make our review more feasible, the impacts of cool roofs on health and wellbeing had to be explicitly measured in the included papers. This meant that papers that discussed the relationship between temperature and health and wellbeing but reported solely on the impacts of cool roofs on temperature were excluded, despite the discussions suggesting the health and wellbeing implications of their findings. Despite their exclusion, these papers likely contribute to understanding the health and wellbeing implications of cool roofs across various settings and expand the breadth of understanding of their effects.

Likewise, to make our review more feasible, we had a restrictive search strategy that may have missed some potentially eligible publications, and we did not search the grey literature or non-English publications. There were also terminology challenges in defining cool roofs, where even with an expansive use of synonyms, complete capture of all synonyms was unlikely. Limiting the Google Scholar search to the first 100 results per query also risked missing relevant studies that did not match the query’s exact wording. Restricting the search to household applications of cool roofs may have missed studies that addressed the health and wellbeing effects of wider community cool roof application. Despite high congruity, screening only 10% of all records by the co-author may have risked excluding borderline studies without a second opinion, especially given the challenges in judging whether health outcomes were explicitly measured. Despite these limitations affecting the potential exclusion of relevant studies, our findings regarding the paucity of empirical evidence and sparse assessment of the contribution of indoor household temperatures, particularly in structurally vulnerable populations, are still likely valid, especially given that these studies were targeted by our search strategy such that relevant articles would likely have been identified.

Third, the quality appraisal was conducted by a single reviewer and involved qualitative judgment. Because the included studies were heterogeneous modelling studies, conventional risk-of-bias tools were not appropriate, and the appraisal framework was adapted from ISPOR-SMDM modelling good-practice principles and tailored to the cool roof health outcome assessments [31]. Although this enabled structured assessment of model credibility, uncertainty and transparency, the ratings should not be interpreted as formal risk-of-bias judgements. Some classifications necessarily involved subjective interpretation, particularly where studies met mixed criteria within domains. For example, models were frequently strongest in their temperature modelling but lacked consistency in carrying this rigour through to downstream health outcomes.

## Conclusions

While our scoping review highlights the potential benefits of cool roofs through modelling studies, there is a dearth of publications that use empirical evidence to link cool roofs to indoor temperatures and related health and wellbeing outcomes, particularly in LMIC and low-resource communities. Research focusing on the health and wellbeing effects of cool roofs on household residents is needed to determine their impact in real-world settings, assess their suitability as a climate change adaptation intervention for heat resilience and address global environmental health inequities. With rising global temperatures and increasing severity and frequency of heat waves, empirical evidence of cool roof effectiveness on resident health and wellbeing in a variety of settings is urgently needed to support effective investment in climate-resilient housing.

## Supplementary Information

Supplementary Material 1.

## Data Availability

No datasets were generated or analysed during the current study.

## Appendix

### Search strategy

Medline (OVID) example:

1. Cool roof (AND a-b) - 46702
  A. Cool (OR i-v) - 476242
    i. Cool.mp. - 12104
    ii. Albedo.mp. - 1877
    ii. White.mp. - 462818
    iv. “Solar reflectance”.mp. - 94
    v. “Thermal emittance”.mp. - 65
  B. Roof (OR i-iv) - 3064424
    i. Roof$.mp. - 10484
    ii. Surface$.mp. - 1563241
    iii. Material$.mp. - 1698667
    iv. Coating$.mp. - 92293
2. Heat (OR a-e) - 1431806
  - Exp. hot temperature/ - 126007
  - Heat$.mp. – 374529
  - Hot$.mp. – 260223
  - Warm$.mp – 93825
  - Temperature$.mp. - 1050169
3. Housing (OR a-e) - 1340158
  - Housing/ - 20272
  - Hous$.mp. – 304416
  - Exp. Home Environment/
  - Home$.mp. – 745213
  - Dwelling$.mp. - 47090
  - Residen$.mp. – 372621
4. AND 1, 2 and 3 – 257

Embase:

AND 1, 2 and 3 = 447.

Scopus:

(Roof* OR surface* OR material* OR coating*) AND (Cool OR Albedo OR White OR “Solar Reflectance” OR “Thermal emittance”) AND (Heat* OR Hot* OR Warm* OR “High Temperature*”) AND (Hous* OR Home* OR Dwelling* OR Residen*) AND (Health* OR Disease* OR Illness* OR Sick* OR Mortalit* OR Death* OR Morbidit* OR Wellbeing OR Wellbeing OR “well being”) = 204.

Web of Science Core Collection:

(Roof* OR surface* OR material* OR coating*) AND (Cool OR Albedo OR White OR “Solar Reflectance” OR “Thermal emittance”) AND (Heat* OR Hot* OR Warm* OR “High Temperature*”) AND (Hous* OR Home* OR Dwelling* OR Residen*) AND (Health* OR Disease* OR Illness* OR Sick* OR Mortalit* OR Death* OR Morbidit* OR Wellbeing OR Wellbeing OR “well being”).

= 642.

CINAHL Complete:

(Roof# OR surface# OR material# OR coating#) AND (Cool OR Albedo OR White OR “Solar Reflectance” OR “Thermal emittance”) AND (Heat# OR Hot# OR Warm# OR “High Temperature#”) AND (Hous# OR Home# OR Dwelling# OR Residen#) AND (Health# OR Disease# OR Illness# OR Sick# OR Mortalit# OR Death# OR Morbidit# OR Wellbeing OR Wellbeing OR “well being”).

= 4.

Google Scholar.

Cool roof heat house health (218,000 results; therefore, the first 10 pages = the first 100 results).

**Table 3:** Quality criteria

Domain	High quality	Moderate quality	Low quality
Model structure	Model type clearly described and justified Structure appropriate to the research question and decision context Key pathways, states, interactions, or mechanisms explicitly represented Exposure-response functions clearly reported and appropriate for the modelled population Time horizon and model perspective justified Structural assumptions clearly explained	Model structure generally described but justification limited Some important pathways or relationships insufficiently explained Time horizon or perspective included but not well justified Structural assumptions only partially described	Model structure poorly described or unclear Inappropriate model type for the research question Major mechanisms/pathways omitted without explanation Time horizon or analytical perspective absent or unjustified
Assumptions	Major assumptions explicitly stated Assumptions scientifically plausible and internally consistent Assumptions supported by literature, empirical data, or theory Structural simplifications acknowledged and justified	Some assumptions described but incompletely Plausibility generally reasonable but limited justification Important simplifications only partially acknowledged	Key assumptions not reported Assumptions implausible, inconsistent, or unsupported Important simplifications ignored or hidden
Data inputs	Data sources clearly identified and referenced Input parameters derived from high-quality empirical evidence Parameter selection justified Missing data handling described Input uncertainty characterised where relevant	Data sources partially described Some parameters based on assumptions or indirect evidence Limited justification for parameter selection Some uncertainty in inputs acknowledged	Data sources poorly described or absent Parameters appear arbitrary or unjustified Heavy reliance on unsupported assumptions No consideration of input uncertainty
Validation	Internal and/or external validation performed and reported Model outputs compared against observed or independent data Calibration methods clearly described where applicable Model performance limitations discussed	Partial validation reported Limited comparison with empirical data Informal calibration or face-validity assessment only Validation methods incompletely described	No validation reported No comparison with observed data Model credibility assumed rather than tested
Uncertainty analysis	Comprehensive uncertainty analysis conducted Includes probabilistic sensitivity analysis, scenario analysis, or multiple sensitivity approaches Structural and parameter uncertainty considered Uncertainty results clearly presented and interpreted	Limited sensitivity analysis conducted Only one-way or deterministic analyses used Some uncertainty acknowledged but incompletely explored	No sensitivity or uncertainty analysis performed Uncertainty ignored or minimally discussed
Transparency	Model methods described in sufficient detail for replication Equations, logic, or analytic steps clearly reported Key parameters and sources provided Supplementary material, diagrams, or code available where appropriate	General modelling approach described Some important technical details missing Partial reproducibility possible	Insufficient methodological detail Model logic unclear Reproduction not feasible from reported information

**Table 4:** Quality appraisal

Reference	Model structure	Assumptions	Data inputs	Validation	Uncertainty analysis	Transparency
Buchin et al. [37] 2016	Moderate	Low	Moderate	Low	Low	Low
He et al. [45] 2020	High	Moderate	High	Moderate	Moderate	High
Kalkstein et al. [38] 2020	Moderate	Moderate	Moderate	Moderate	Low	Moderate
Liu et al. [44] 2023	Moderate	Moderate	Moderate	Moderate	Low	Moderate
Macintyre and Heaviside [33] 2019	High	Moderate	High	Moderate	Moderate	High
Macintyre et al. [34] 2021	High	Moderate	High	Moderate	Moderate	High
Nutkiewicz et al. [36] 2022	Moderate	Low	Moderate	Low	Moderate	Low
Sailor et al. [35] 2021	High	Moderate	High	Moderate	Low	Moderate
Salata et al. [42] 2017	Moderate	High	High	Moderate	Low	Low
Santamouris et al. [39] 2020	Moderate	Moderate	High	High	Moderate	Low
Stone et al. [40] 2014	Moderate	Moderate	High	Moderate	High	Low
Susca [46] 2012	Moderate	Moderate	Moderate	Low	Low	High
Vargo et al. [41] 2016	Moderate	Moderate	High	Low	Moderate	Low
Wang et al. [43] 2022	Moderate	Moderate	Moderate	Moderate	Low	High

## Declarations

### Use of Artificial Intelligence Tools

No generative artificial intelligence or large language model tools were used in the conception, design, conduct, data extraction, analysis, or writing of this review.

## References

[1] Willett KM, Sherwood S. Exceedance of heat index thresholds for 15 regions under a warming climate using the wet-bulb globe temperature. Int J Climatol. 2012;32(2):161–77. 10.1002/joc.2257.

[2] Dunne JP, Stouffer RJ, John JG. Reductions in labour capacity from heat stress under climate warming. Nat Clim Change. 2013;3(6):563–6. 10.1038/nclimate1827.

[3] Schwingshackl C, Sillmann J, Vicedo-Cabrera A, Sandstad M, Aunan K. Heat stress indicators in CMIP6: estimating future trends and exceedances of impact-relevant thresholds. Earths Future. 2021;9(3). 10.1029/2020EF001885. e2020EF001885.

[4] Wang Y, Zhao N, Wu C, Quan J, Chen M. Future population exposure to heatwaves in 83 global megacities. Sci Total Environ. 2023;888:164142. 10.1016/j.scitotenv.2023.164142. 37182769

[5] Vargas Zeppetello LR, Raftery AE, Battisti DS. Probabilistic projections of increased heat stress driven by climate change. Commun Earth Environ. 2022;3(1):183. 10.1038/s43247-022-00524-4. 39421457 PMC11485542

[6] Mason H, King JC, Peden AE, Franklin RC. Systematic review of the impact of heatwaves on health service demand in Australia. BMC Health Serv Res. 2022;22(1):960. 10.1186/s12913-022-08341-3. 35902847 PMC9336006

[7] Liu J, Varghese BM, Hansen A, Zhang Y, Driscoll T, Morgan G, et al. Heat exposure and cardiovascular health outcomes: a systematic review and meta-analysis. Lancet Planet Health. 2022;6(6):e484–95. 10.1016/S2542-5196(22)00117-6. 35709806

[8] Kovats RS, Hajat S. Heat stress and public health: a critical review. Annu Rev Public Health. 2008;29(1):41–55. 10.1146/annurev.publhealth.29.020907.090843. 18031221

[9] Ebi KL, Capon A, Berry P, Broderick C, de Dear R, Havenith G, et al. Hot weather and heat extremes: health risks. Lancet. 2021;398(10301):698–708. 10.1016/S0140-6736(21)01208-3. 34419205

[10] Bunker A, Wildenhain J, Vandenbergh A, Henschke N, Rocklöv J, Hajat S, et al. Effects of air temperature on climate-sensitive mortality and morbidity outcomes in the elderly: a systematic review and meta-analysis of epidemiological evidence. EBioMedicine. 2016;6:258–68. 10.1016/j.ebiom.2016.02.034. 27211569 PMC4856745

[11] Ige-Elegbede J, Powell J, Pilkington P. A systematic review of the impacts of extreme heat on health and wellbeing in the United Kingdom. Cities Health. 2024;8(3):432–46. 10.1080/23748834.2023.2283240.

[12] Zhao Q, Guo Y, Ye T, Gasparrini A, Tong S, Overcenco A, et al. Global, regional, and national burden of mortality associated with non-optimal ambient temperatures from 2000 to 2019: a three-stage modelling study. Lancet Planet Health. 2021;5(7):e415–25. 10.1016/S2542-5196(21)00081-4. 34245712

[13] Romanello M, Walawender M, Hsu SC, Moskeland A, Palmeiro-Silva Y, Scamman D, et al. The 2025 report of the Lancet Countdown on health and climate change: climate change action offers a lifeline. Lancet. 2025;406(10521):2804–57. 10.1016/S0140-6736(25)01919-1. 41175887

[14] McIver L, Kim R, Woodward A, Hales S, Spickett J, Katscherian D, et al. Health impacts of climate change in Pacific Island countries: a regional assessment of vulnerabilities and adaptation priorities. Environ Health Perspect. 2016;124(11):1707–14. 10.1289/ehp.1509756. 26645102 PMC5089897

[15] Levinson R, Akbari H. Potential benefits of cool roofs on commercial buildings: conserving energy, saving money, and reducing emission of greenhouse gases and air pollutants. Energy Effic. 2010;3(1):53–109. 10.1007/s12053-008-9038-2.

[16] Manoli G, Fatichi S, Schläpfer M, Yu K, Crowther TW, Meili N, et al. Magnitude of urban heat islands largely explained by climate and population. Nature. 2019;573(7772):55–60. 10.1038/s41586-019-1512-9. 31485056

[17] Wouters H, De Ridder K, Poelmans L, Willems P, Brouwers J, Hosseinzadehtalaei P, et al. Heat stress increase under climate change twice as large in cities as in rural areas: a study for a densely populated midlatitude maritime region. Geophys Res Lett. 2017;44(17):8997–9007. 10.1002/2017GL074889.

[18] Kolokotroni M, Shittu E, Santos T, Ramowski L, Mollard A, Rowe K, et al. Cool roofs: high-tech low-cost solution for energy efficiency and thermal comfort in low-rise low-income houses in high solar radiation countries. Energy Build. 2018;176:58–70. 10.1016/j.enbuild.2018.07.005.

[19] Brousse O, Simpson C, Zonato A, Martilli A, Taylor J, Davies M, et al. Cool roofs could be most effective at reducing outdoor urban temperatures in London, United Kingdom, compared with other rooftop and vegetation interventions: a mesoscale urban climate modeling study. Geophys Res Lett. 2024;51(13):e2024GL109634. 10.1029/2024GL109634.

[20] Akbari H, Levinson R, Rainer L. Monitoring the energy-use effects of cool roofs on California commercial buildings. Energy Build. 2005;37(10):1007–16. 10.1016/j.enbuild.2004.11.013.

[21] Xu X, Chen F, Shen S, Miao S, Barlage M, Guo W, et al. Using WRF-Urban to assess summertime air conditioning electric loads and their impacts on urban weather in Beijing. J Geophys Res Atmos. 2018;123(5):2475–90. 10.1002/2017JD028168.

[22] Ashtari B, Yeganeh M, Bemanian M, Vojdani Fakhr B. A conceptual review of the potential of cool roofs as an effective passive solar technique: elaboration of benefits and drawbacks. Front Energy Res. 2021;9:738182. 10.3389/fenrg.2021.738182.

[23] Elnabawi MH, Alhumaidi A, Osman B, Alshehhi R. Cool roofs in hot climates: a conceptual review of modelling methods and limitations. Buildings. 2022;12(11):1968. 10.3390/buildings12111968.

[24] Santamouris M, Synnefa A, Karlessi T. Using advanced cool materials in the urban built environment to mitigate heat islands and improve thermal comfort conditions. Sol Energy. 2011;85(12):3085–102. 10.1016/j.solener.2010.12.023.

[25] Testa J, Krarti M. A review of benefits and limitations of static and switchable cool roof systems. Renew Sustain Energy Rev. 2017;77:451–60. 10.1016/j.rser.2017.04.030.

[26] Tricco AC, Soobiah C, Antony J, Cogo E, MacDonald H, Lillie E, et al. A scoping review identifies multiple emerging knowledge synthesis methods, but few studies operationalize the method. J Clin Epidemiol. 2016;73:19–28. 10.1016/j.jclinepi.2015.08.030. 26891949

[27] Arksey H, O’Malley L. Scoping studies: towards a methodological framework. Int J Soc Res Methodol. 2005;8(1):19–32. 10.1080/1364557032000119616.

[28] Tricco AC, Lillie E, Zarin W, O’Brien KK, Colquhoun H, Levac D, et al. PRISMA extension for scoping reviews (PRISMA-ScR): checklist and explanation. Ann Intern Med. 2018;169(7):467–73. 10.7326/M18-0850. 30178033

[29] Ouzzani M, Hammady H, Fedorowicz Z, Elmagarmid A. Rayyan: a web and mobile app for systematic reviews. Syst Rev. 2016;5(1):210. 10.1186/s13643-016-0384-4. 27919275 PMC5139140

[30] McDonagh M, Peterson K, Raina P, Chang S, Shekelle P. Avoiding bias in selecting studies. Methods Guide for Effectiveness and Comparative Effectiveness Reviews. Rockville, MD: Agency for Healthcare Research and Quality; 2008. 23487864

[31] Caro JJ, Briggs AH, Siebert U, Kuntz KM, ISPOR-SMDM Modeling Good Research Practices Task Force. Modeling good research practices: overview: a report of the ISPOR-SMDM Modeling Good Research Practices Task Force-1. Value Health. 2012;15(6):796–803. 10.1016/j.jval.2012.06.012. 22999128

[32] Bennett C, Manuel DG. Reporting guidelines for modelling studies. BMC Med Res Methodol. 2012;12(1):168. 10.1186/1471-2288-12-168. 23134698 PMC3533955

[33] Macintyre HL, Heaviside C. Potential benefits of cool roofs in reducing heat-related mortality during heatwaves in a European city. Environ Int. 2019;127:430–41. 10.1016/j.envint.2019.02.065. 30959308

[34] Macintyre HL, Heaviside C, Cai XM, Phalkey R. Comparing temperature-related mortality impacts of cool roofs in winter and summer in a highly urbanized European region for present and future climate. Environ Int. 2021;154:106606. 10.1016/j.envint.2021.106606. 33971480 PMC8214226

[35] Sailor DJ, Anand J, Kalkstein L. Potential overall heat exposure reduction associated with implementation of heat mitigation strategies in Los Angeles. Int J Biometeorol. 2021;65(3):407–18. 10.1007/s00484-020-01954-5. 32562041

[36] Nutkiewicz A, Mastrucci A, Rao ND, Jain RK. Cool roofs can mitigate cooling energy demand for informal settlement dwellers. Renew Sustain Energy Rev. 2022;159:112183. 10.1016/j.rser.2022.112183.

[37] Buchin O, Hoelscher M, Meier F, Nehls T, Ziegler F. Evaluation of the health-risk reduction potential of countermeasures to urban heat islands. Energy Build. 2016;114:27–37. 10.1016/j.enbuild.2015.06.038.

[38] Kalkstein L, Klink F, Shickman K, Schneider S, Egolf M, Sailor D. The potential impact of cool roof technologies upon heat wave meteorology and human health in Boston and Chicago. Roofing Research and Standards Development. Volume 9. West Conshohocken, PA: ASTM International; 2020. pp. 1–27. 10.1520/STP162120180127.

[39] Santamouris M, Paolini R, Haddad S, Synnefa A, Garshasbi S, Hatvani-Kovacs G, et al. Heat mitigation technologies can improve sustainability in cities: a holistic experimental and numerical impact assessment of urban overheating and related heat mitigation strategies on energy consumption, indoor comfort, vulnerability and heat-related mortality and morbidity in cities. Energy Build. 2020;217:110002. 10.1016/j.enbuild.2020.110002.

[40] Stone B Jr, Vargo J, Liu P, Habeeb D, DeLucia A, Trail M, et al. Avoided heat-related mortality through climate adaptation strategies in three US cities. PLoS ONE. 2014;9(6):e100852. 10.1371/journal.pone.0100852. 24964213 PMC4071007

[41] Vargo J, Stone B, Habeeb D, Liu P, Russell A. The social and spatial distribution of temperature-related health impacts from urban heat island reduction policies. Environ Sci Policy. 2016;66:366–74. 10.1016/j.envsci.2016.08.012.

[42] Salata F, Golasi I, Petitti D, de Lieto Vollaro E, Coppi M, de Lieto Vollaro A. Relating microclimate, human thermal comfort and health during heat waves: an analysis of heat island mitigation strategies through a case study in an urban outdoor environment. Sustain Cities Soc. 2017;30:79–96. 10.1016/j.scs.2017.01.006.

[43] Wang X, Li H, Sodoudi S. The effectiveness of cool and green roofs in mitigating urban heat island and improving human thermal comfort. Build Environ. 2022;217:109082. 10.1016/j.buildenv.2022.109082.

[44] Liu S, Levinson R, Narumi D. Numerical analysis of various heat countermeasures: effects on energy consumption and indoor thermal comfort in densely built wooden house area. Atmosphere. 2023;14(10):1566. 10.3390/atmos14101566.

[45] He C, He L, Zhang Y, Kinney PL, Ma W. Potential impacts of cool and green roofs on temperature-related mortality in the Greater Boston region. Environ Res Lett. 2020;15(9):094042. 10.1088/1748-9326/aba4c9.

[46] Susca T. Multiscale approach to life cycle assessment. J Ind Ecol. 2012;16(6):951–62. 10.1111/j.1530-9290.2012.00560.x.

[47] Shi L, Kloog I, Zanobetti A, Liu P, Schwartz JD. Impacts of temperature and its variability on mortality in New England. Nat Clim Change. 2015;5(11):988–91. 10.1038/nclimate2704. PMC4666547 26640524

[48] Gagge AP, Stolwijk JAJ, Nishi Y. An effective temperature scale based on a simple model of human physiological regulatory response. ASHRAE Trans. 1971;77:247–62.

[49] Vardoulakis S, Dear K, Hajat S, Heaviside C, Eggen B, McMichael AJ. Comparative assessment of the effects of climate change on heat- and cold-related mortality in the United Kingdom and Australia. Environ Health Perspect. 2014;122(12):1285–92. 10.1289/ehp.1307524. 25222967 PMC4256046

[50] Sherwood SC, Huber M. An adaptability limit to climate change due to heat stress. Proc Natl Acad Sci U S A. 2010;107(21):9552–5. 10.1073/pnas.0913352107. 20439769 PMC2906879

[51] Baccini M, Biggeri A, Accetta G, Kosatsky T, Katsouyanni K, Analitis A, et al. Heat effects on mortality in 15 European cities. Epidemiology. 2008;19(5):711–9. 10.1097/EDE.0b013e318176bfcd. 18520615

[52] Medina-Ramon M, Schwartz J. Temperature, temperature extremes, and mortality: a study of acclimatisation and effect modification in 50 US cities. Occup Environ Med. 2007;64(12):827–33. 10.1136/oem.2007.033175. 17600037 PMC2095353

[53] Zanobetti A, Schwartz J. Temperature and mortality in nine US cities. Epidemiology. 2008;19(4):563–70. 10.1097/EDE.0b013e31816d652d. 18467963 PMC3722554

[54] Anderson GB, Bell ML. Heat waves in the United States: mortality risk during heat waves and effect modification by heat wave characteristics in 43 US communities. Environ Health Perspect. 2011;119(2):210–8. 10.1289/ehp.1002313. 21084239 PMC3040608

[55] Metzger KB, Ito K, Matte TD. Summer heat and mortality in New York City: how hot is too hot? Environ Health Perspect. 2010;118(1):80–6. 10.1289/ehp.0900906. 20056571 PMC2831972

[56] Cole BL, Fielding JE. Health impact assessment: a tool to help policy makers understand health beyond health care. Annu Rev Public Health. 2007;28:393–412. 10.1146/annurev.publhealth.28.083006.131942. 17173539

[57] Kenny GP, Tetzlaff EJ, Journeay WS, Henderson SB, O’Connor FK. Indoor overheating: a review of vulnerabilities, causes, and strategies to prevent adverse human health outcomes during extreme heat events. Temperature. 2024;11(3):203–46. 10.1080/23328940.2024.2361223. PMC11346563 39193048

[58] Xia X, Chan KH, Niu Y, Liu C, Guo Y, Ho K, et al. Modelling personal temperature exposure using household and outdoor temperature and questionnaire data: implications for epidemiological studies. Environ Int. 2024;192:109060. 10.1016/j.envint.2024.109060. 39401479 PMC7616742

[59] Fung KY, Yang Z, Martilli A, Krayenhoff ES, Niyogi D. Prioritizing social vulnerability in urban heat mitigation. PNAS Nexus. 2024;3(9):pgae360. 10.1093/pnasnexus/pgae360. 39262852 PMC11388001

[60] Cravioto J, Mosqueda A. Local culture and urban retrofit: reflections on policy and preferences for wall and roof materials. Front Sustain Cities. 2021;3:638966. 10.3389/frsc.2021.638966.

[61] Kolokotsa D, Diakaki C, Papantoniou S, Vlissidis A. Numerical and experimental analysis of cool roofs application on a laboratory building in Iraklion. Crete Greece Energy Build. 2012;55:85–93. 10.1016/j.enbuild.2011.09.011.

[62] Das JK, Kumari B, Rahman AR, Azhar M, Khan S, Masood U, et al. Evaluation of community-based heat adaptation interventions: a systematic review. BMJ Public Health. 2025;3(2):e002332. 10.1136/bmjph-2024-002332. 40687959 PMC12273142

[63] Yang Y, Pan Z, Zhang B, Huang S, Chen X, Hong T. Review of cooling effects from roof mitigation strategies against urban heat island effects. Buildings. 2025;15(21):3835. 10.3390/buildings15213835.

[64] Johar H, Abdulsalam FI, Guo Y, Baernighausen T, Jahan NK, Watterson J, et al. Community-based heat adaptation interventions for improving heat literacy, behaviours, and health outcomes: a systematic review. Lancet Planet Health. 2025;9(7):101207. 10.1016/S2542-5196(25)00007-5. 40258380

[65] Zheng Z, Fong CS, Aghamohammadi N, Law YK. A systematic review of urban heat island impacts and mitigation: health, equity, and policy. Systems. 2026;14(1):82. 10.3390/systems14010082.

[66] Metcalf CJE, Edmunds WJ, Lessler J. Six challenges in modelling for public health policy. Epidemics. 2015;10:93–6. 10.1016/j.epidem.2014.08.008. 25843392

[67] Siddiqui SA, Thiam S, Houndodjade C, Murage P, Bonell A. A systematic review and meta-analysis of the impact of environmental heat exposure on cardiovascular diseases, chronic respiratory diseases and diabetes mellitus in low- and middle-income countries. Environ Res. 2025;282:121980. 10.1016/j.envres.2025.121980. 40451411

[68] Cheng J, Xu Z, Bambrick H, Prescott V, Wang N, Zhang Y, et al. Cardiorespiratory effects of heatwaves: a systematic review and meta-analysis of global epidemiological evidence. Environ Res. 2019;177:108610. 10.1016/j.envres.2019.108610. 31376629

[69] Arsad FS, Hod R, Ahmad N, Ismail R, Mohamed N, Baharom M, et al. The impact of heatwaves on mortality and morbidity and the associated vulnerability factors: a systematic review. Int J Environ Res Public Health. 2022;19(23):16356. 10.3390/ijerph192316356. 36498428 PMC9738283

[70] Jay O, Capon A, Berry P, Broderick C, de Dear R, Havenith G, et al. Reducing the health effects of hot weather and heat extremes: from personal cooling strategies to green cities. Lancet. 2021;398(10301):709–24. 10.1016/S0140-6736(21)01209-5. 34419206

[71] Hariton E, Locascio JJ. Randomised controlled trials: the gold standard for effectiveness research. BJOG. 2018;125(13):1716. 10.1111/1471-0528.15199. 29916205 PMC6235704

[72] Bunker A, Compoaré G, Sewe MO, Cedeno Laurent JG, Zabré P, Boudo V, et al. The effects of cool roofs on health, environmental, and economic outcomes in rural Africa: study protocol for a community-based cluster randomized controlled trial. Trials. 2024;25(1):59. 10.1186/s13063-023-07804-0. 38229177 PMC10792891

[73] University of Auckland. REFLECT: a global heat adaptation study. University of Auckland; 2025. https://www.reflect.org.nz/. Accessed 10 Jun 2025.

[74] Institute of Development Studies. REFLECT: a global heat adaptation study. HeatNexus; 2026. https://heatnexus.org/projects/reflect-a-global-heat-adaptation-study/. Accessed 19 May 2026.

